# Host Jump of an Exotic Fish Rhabdovirus into a New Class of Animals Poses a Disease Threat to Amphibians

**DOI:** 10.3390/v16081193

**Published:** 2024-07-25

**Authors:** Eveline J. Emmenegger, Emma K. Bueren, Carla M. Conway, George E. Sanders, A. Noble Hendrix, Tamara Schroeder, Emiliano Di Cicco, Phuc H. Pham, John S. Lumsden, Sharon C. Clouthier

**Affiliations:** 1 U.S. Geological Survey, Western Fisheries Research Center (WFRC), 6505 NE 65th Street, Seattle, WA 98115, USA; 2 Department of Biology, Indiana University, 1001 E 3rd St, Bloomington, IN 47405, USA; 3 Department of Comparative Medicine, University of Washington, Seattle, WA 98195, USA; 4 QEDA Consulting, 4007 Densmore Avenue N, Seattle, WA 98103, USA; 5 School of Aquatic and Fishery Sciences, University of Washington, Seattle, WA 98195, USA; 6 Freshwater Institute, Fisheries and Oceans Canada (DFO), 501 University Crescent, Winnipeg, MB R3T 2N6, Canada; 7 Pacific Salmon Foundation (PSF), 1682 W 7th Ave., Vancouver, BC V6J 4S6, Canada; 8 Department of Pathobiology, Ontario Veterinary College, University of Guelph, Guelph, ON N1G 2W1, Canada

**Keywords:** spring viremia of carp virus (SVCV), host jump, cross-species, amphibian, RNA virus, rhabdovirus, invasive species, disease threat, spillover

## Abstract

Spring viremia of carp virus (SVCV) is a rhabdovirus that primarily infects cyprinid finfishes and causes a disease notifiable to the World Organization for Animal Health. Amphibians, which are sympatric with cyprinids in freshwater ecosystems, are considered non-permissive hosts of rhabdoviruses. The potential host range expansion of SVCV in an atypical host species was evaluated by testing the susceptibility of amphibians native to the Pacific Northwest. Larval long-toed salamanders *Ambystoma macrodactylum* and Pacific tree frog *Pseudacris regilla* tadpoles were exposed to SVCV strains from genotypes Ia, Ib, Ic, or Id by either intraperitoneal injection, immersion, or cohabitation with virus-infected koi *Cyprinus rubrofuscus*. Cumulative mortality was 100% for salamanders injected with SVCV, 98–100% for tadpoles exposed to virus via immersion, and 0–100% for tadpoles cohabited with SVCV-infected koi. Many of the animals that died exhibited clinical signs of disease and SVCV RNA was found by in situ hybridization in tissue sections of immersion-exposed tadpoles, particularly in the cells of the gastrointestinal tract and liver. SVCV was also detected by plaque assay and RT-qPCR testing in both amphibian species regardless of the virus exposure method, and viable virus was detected up to 28 days after initial exposure. Recovery of infectious virus from naïve tadpoles cohabited with SVCV-infected koi further demonstrated that SVCV transmission can occur between classes of ectothermic vertebrates. Collectively, these results indicated that SVCV, a fish rhabdovirus, can be transmitted to and cause lethal disease in two amphibian species. Therefore, members of all five of the major vertebrate groups (mammals, birds, reptiles, fish, and amphibians) appear to be vulnerable to rhabdovirus infections. Future research studying potential spillover and spillback infections of aquatic rhabdoviruses between foreign and domestic amphibian and fish species will provide insights into the stressors driving novel interclass virus transmission events.

## 1. Introduction

RNA viruses are known for rapid emergence and disease epidemics in novel host species [1]. The fitness of these viruses is based less on co-evolution with their host and more on their ability to generate a pool of variants with greater genetic diversity. The high mutation rate of their RNA-dependent RNA polymerases during virus replication can facilitate cross-species infections or host jumps [2,3]. A staggering number of RNA viruses are being discovered via metagenomic analyses with a large proportion found in fish species. These RNA fish viruses are rudimentary to the evolution of RNA viruses of terrestrial hosts and host switching by vertebrate RNA viruses occurs often among host species who share the same habitat [4], such as aquatic environments. 

Rhabdoviruses are RNA viruses that are bullet-shaped, have a single stranded negative-sense RNA genome, and are known to have the highest frequency of cross-species transmission among vertebrate hosts [1,5]. The natural hosts of rhabdoviruses are ecologically diverse and include plants, insects, reptiles, mammals, and fish [2,6]. They are among the most numerous aquatic viruses to be found in association with fish die-off events [7,8]. Amphibians share habitat with fish as a consequence of their aquatic life stages, yet they are not known to be natural host species of extant rhabdoviruses.

Aquatic rhabdoviruses are taxonomically classified into six genera: *Novirhabdovirus* (subfamily: *Gammarhabdovirinae*), *Cetarhavirus*, *Perhabdovirus*, *Scophrhavirus*, *Siniperhavirus*, and *Sprivivirus* [9]. The fish novirhabdoviruses are the basal species in the evolution of the *Rhabdoviridae* family [10,11]. The five remaining aquatic rhabdovirus genera (*Cetarhavirus*, *Perhabdovirus*, *Scophrhavirus*, *Siniperhavirus*, and *Sprivivirus*) have diverged together and are more closely related to rhabdoviruses infecting terrestrial and mammalian host species [12]. Rhabdoviruses from these five genera infect cetaceans and fish and have the five canonical rhabodovirus genes arranged in their genome as N-P-M-G-L (N, nucleoprotein; P, phosphoprotein; M, matrix; G, glycoprotein; L, polymerase) [11,13]. 

*Carpio sprivivirus*, commonly known as spring viremia of carp virus (SVCV), is a temperate/warm-water fish rhabdovirus of the *Sprivivirus* genus, which is the most closely related fish rhabdovirus genus to the mammalian *Vesiculovirus* genus (subfamily: *Alpharhabdovirinae*) [9,11,14]. The primary host species of SVCV is the common carp *Cyprinus carpio* Linneaeus 1758 [15]. Koi, *Cyprinus rubrofuscus* Lacepède 1803, also known as *Cyprinus carpio haematopterus*, *Cyprinus carpio koi*, or Nishikigoi, is a domesticated version of Amur carp with numerous ornamental hybrid carp varieties that are also highly susceptible to SVCV [15,16,17,18]. Many other fish species from the Cyprinidae family are considered vulnerable to infection and disease by SVCV [19]. There have been some reports describing detections and/or outbreaks of SVCV in seven additional fish families: Acipenseridae, Catostomidae, Centrarchidae, Cichlidae, Escocidae, Salmonidae, and Siluridae [19,20,21,22,23,24,25,26,27]. The virus has also been isolated from diseased penaeid shrimp, farmed in the Hawaiian Islands [28,29]. 

SVCV outbreaks in the primary hosts (common carp and koi) typically occur in the spring as water temperatures rise after a cold winter and occur both in wild and cultured fish [15]. Adults and young fish alike can succumb to SVC disease, but fry and juveniles less than a year old are most susceptible [30,31]. Infected fish exhibit numerous clinical signs of signs of disease, including pale gills, bloody ascites fluid, exopthalmia, and diffuse hemorrhaging [32]. The virus was first described in carp from the former European country of Yugoslavia in the 1970s and now SVC disease is considered well-established throughout Europe with epidemics primarily occurring in carp farms [15,33,34]. The virus was later found in fish species from countries within South America (Brazil) and North America (United States, Canada, Mexico) starting in 1998 and 2002, respectively [35,36,37,38,39]. Detections of SVCV in diseased and asymptomatic fish were first reported by China and South Korea starting in 2004 [25,40]. Suspected cases of SVCV in fish from the Middle East (Iran) and Africa (Egypt) were reported in 2008 [20,41,42]. 

Genetic typing of SVCV isolates groups them into four genotypes, Ia, Ib, Ic, and Id, that are correlated with geographic origin [43,44]. Strains of genotype Ib, Ic, and Id originated from central and eastern European countries. The Ia isolates are of Asian origin and thus far all confirmed discrete SVCV isolations within North America belong to this genogroup [27,38,45,46]. The World Organization of Animal Health (WOAH) and the U.S. Department of Agriculture’s Animal and Plant Health Inspection Service (USDA APHIS) categorizes SVCV as an exotic pathogen of a mandatory reportable disease when detected in North America [19,47]. Since the mid-1990s, there has been a resurgence of SVCV genotype Ia and Id infections in North America and Europe, respectively, with genogroup Ia variants estimated to have gene nucleotide substitution rates 3–4 times higher than genotype Id strains [48]. Concomitantly, outbreaks and isolations of exotic SVCV (genotype Ia isolates) occurred in new fish species, Bluegill *Lepomis macrochirus* (*Centrarchidae*), Largemouth bass *Micropterus salmoides* (*Centrarchidae*), and Quillback Sucker *Carpiodes cyprinusis* (*Catostomidae*), that are native to North American watersheds and are not from the *Cyprinidae* family [27,46]. These findings may indicate that novel host jumps are occurring in tandem with the re-emergence of SVCV genotype Ia strains. 

Wildlife and domestic animal trade activities have been linked to virus spillover transmissions into novel hosts with subsequent epidemics or pandemics occurring in naïve atypical hosts (e.g., avian influenza (H5N1 bird flu), SARS-CoV-2 (COVID-19)) [49,50,51,52]. The global movement of animal products and species for commerce, including within the aquaculture and ornamental aquatic pet industries, is thought to be the origin of many emerging aquatic animal diseases. The breadth of viral pathogens that could be threatening amphibian populations is only starting to be revealed [53,54,55,56,57,58,59,60]. Chinese fire-bellied newts *Cynops orientalis* are a popular ornamental salamander reared in home aquariums and one of the most globally traded pet amphibian species entering the United States [61,62]. An unexpected isolation of SVCV from *C. orientalis* imported into the state of Florida (USA) occurred in 2015 [63]. The newts were shipped from Hong Kong and appeared unhealthy or dead upon their arrival in the USA. The initial concern was that the newts were infected with the chytrid fungus *Batrachochytrium salamandrivorans* (Bsal), which has decimated salamander populations globally [64]. Diagnostic testing revealed that they were infected with an Asian genotype Ia SVCV strain [63]. This was the first time a rhabdovirus had been associated with disease in an amphibian species. 

To determine if the initial SVCV detection in an amphibian species was an anomalous pathogen spillover event or if this fish rhabdovirus can infect and cause disease in species from the class Amphibia, which are considered non-permissive hosts, we exposed two amphibian species native to the Pacific Northwest of North America to various SVCV strains by three routes of exposure (injection, immersion, and cohabitation). This study provides a preliminary assessment of amphibian species susceptibility to the exotic SVCV and explores the possibility that a novel viral cross-species host jump has occurred from fish into amphibians.

## 2. Materials and Methods

### 2.1. Native Amphibian Host Species 

Larval Western Long-toed salamanders *Ambystoma macrodactylum* with average weights of 1.1 g (0.7–1.38 g) and average lengths of 6.5 cm (5.7–7.3 cm) and Pacific tree frog *Pseudacris regilla* tadpoles with average weights of 0.28 g (0.8–0.61 g) and average lengths of 1.9 cm (1.0–3.0 cm) were manually collected via dip net from a spring-fed private pond in Kenmore, Washington. The wild native amphibians were transferred to the Western Fisheries Research Center (WFRC) biosafety level -2 (BSL-2) main wet laboratory in Seattle, Washington. The animals were quarantined for two weeks upon arrival by segregation from other fish species present at the facility and remained healthy until the initiation of each challenge experiment. The collected tadpoles were reared in two separate 4-foot wide circular tanks. Each larval salamander was individually housed to avoid cohort aggression in a 3 L tank containing an enrichment PVC tunnel. If any of the larval salamanders progressed to the metamorph stage, haul-out structures were also placed in the tanks. Single-pass, sand-filtered, and UV-treated freshwater from Lake Washington flowed into the stock animal tanks at temperatures of 16.5 °C for salamanders and 13–14 °C for tadpoles. The animals were fed every other day. Salamanders received frozen blood worms and brine shrimp (Hakari Inc., Kyorin Food Industries, Tokyo, Japan) and tadpoles were provided pieces of frozen boiled organic lettuce along with Aquatic Frog and Tadpole Food micro pellets (Zoo Med Laboratories, San Luis Obispo, CA, USA). Standard operating procedures for rearing live aquatic animals and research protocols utilized in the amphibian challenge experiments were approved by the Institutional Animal Care and Use Committee (IACUC) of the WFRC under the guidelines provided by the Guide for the Care and Use of Laboratory Animals [65] and the American Veterinary Medical Association [66].

### 2.2. Virus Strains

A total of six SVCV strains (20040741, 20070165, NC2002, RHV, P4-7, and Fijan), with representatives from each genotype (Ia, Ib, Ic, and Id), were tested in three infection susceptibility experiments. The source features of each SVCV isolate are listed in Table 1. Hereafter, any abbreviation listed as ‘Ia’, ‘Ib’, ‘Ic’, or ‘Id’ refers to the genotype of an SVCV isolate. Virus isolates and propagation methods were previously described in [18]. To create the virus stocks for this study, virus isolates were cultured at 20 °C in flasks containing confluent monolayers of epithelioma papulosum cyprini cells and then harvested after the cell monolayer displayed significant cytopathic effect (CPE) [67,68,69]. The clarified supernatants of each SVCV strain were frozen at −80 °C, the virus titer of a thawed virus aliquot was quantified via plaque assay, and the freezer stocks were utilized in the virus challenge experiments described herein. Virus titer was expressed as plaque-forming units (PFU)/mL [70,71].

### 2.3. Injection Challenge Experiment of Western Long-Toed Salamanders

On the day of challenge, larval salamanders were transferred to the aquatic biosafety level-3 (BSL-3) laboratory which is the required laboratory biocontainment level for SVCV in vivo experiments in the United States. The salamanders were arbitrarily separated into 2 virus treatment groups or a negative control group with 4 replicate tanks in total per treatment (1 salamander/tank). Each animal was anesthetized in a 1 L water bath containing 1.0 g of tricaine methanesulfonate (MS-222, Syndel, Ferndale, WA, USA) and 1.0 g sodium bicarbonate (NaHCO_3_) prior to being intraperitoneally injected with a 50 µL inoculum. Virus-exposed salamanders each received an injection of SVCV isolate 20070165 or 20040741 (both genotype Ia strains) at a dose of 4.3 × 10^6^ or 2.3 × 10^6^ PFU/salamander, respectively. The salamanders in the negative control group each received an injection of cell culture media consisting of minimal essential medium (Thermo Scientific, Waltham, MA, USA) supplemented with 10% fetal bovine serum and buffered with sodium bicarbonate (MEM-10-SB). After injection, individual salamanders were returned to a 3 L tank receiving low-flow through water set at 11 °C on the day of challenge (Day 0), raised 1 °C/day until water temperatures reached 14 °C (Day 3), and then held constant until the end of the experiment (Day 20). Susceptibility of the larval salamanders to SVCV was assessed via cumulative mortality. The experiment water temperature profile was selected to simulate conditions of natural SVCV outbreaks that typically occur as water temperatures rise in the spring. This approach was adapted from SVCV challenge experiments that resulted in infection and disease in finfish species [68,73,74]. The salamanders were monitored daily and fed every other day. Severely moribund salamanders were euthanized using a lethal solution of 0.5% MS-222 (5.0 g in 1 L buffered with 5.0 g NaHCO_3_), and dead salamanders were removed from tanks on day of death. Whole animal specimens were placed individually in a Whirl-Pak bag (Nasco Sampling LLC, Pleasant Prairie, WI, USA), and immediately frozen at −80 °C until processed for virus detection. 

### 2.4. Immersion Challenge Experiment of Pacific Tree Frog Tadpoles

Pacific tree frog tadpoles were transferred to the aquatic BSL-3 laboratory on the day of challenge (Day 0) and arbitrarily divided into one of the five virus treatment groups (SVCV strains: 20040741, NC2002, RHV, P4-7, or Fijan) or the negative control treatment group. Animals were exposed to a specific SVCV isolate in 3 L of static water at an exposure a dose of 1 × 10^5^ PFU/L for 1 h, after which continuous flow-through water in a 6 L volume was resumed. Water temperature was held constant at 14.3–14.5 °C throughout the 28-day experiment with one exception. Water temperatures rose to a maximum of 16 °C for a 30 min period on day 22 post-exposure when water flow ceased for 1.5 h due to mechanical issues with the BSL-3 lab water supply system. Tadpoles in the negative control treatment group were exposed to an equivalent volume of cell culture medium in lieu of virus. Henceforth, animals from negative control treatments will also be referred to as mock-exposed or from mock treatments. All animals were observed daily and fed every other day. Cumulative mortality was assessed for 28 days using 3 replicate tanks/treatment, where each tank contained between 16 and 22 tadpoles. Animals were removed on day of death and/or euthanized if they exhibited excessive morbidity. Targeted sampling of an additional tank/treatment (*n* = 25 tadpoles/tank) was included for a semiquantitative assessment of infection dynamics. In this case, 3 tadpoles per tank were removed at 2, 4, 6, 8, 11, 15, and 18 days post-exposure. These animals were euthanized in a buffered solution of 0.5% MS 222. At the end of the experiment (Day 28), any survivors from these tanks were euthanized and sampled. Most of the collected specimens consisted of single tadpoles placed in individual Whirl-Pak bags. During the immersion challenge, if two tadpoles died in the same replicate treatment tank on the same day and were excessively small (≤1.0 cm), they were pooled together, frozen in the same bag, weighed together, and processed as a single specimen for virus detection. The presence of any external clinical signs of disease on the collected specimens were recorded and the tadpoles were frozen at −80 °C until samples were processed. 

### 2.5. Histopathology of Tadpoles from Immersion Challenge

Tadpoles for histopathological examination were removed 7 or 8 days following immersion challenge with SVCV NC2002, 20040741, P4-7, or Fijan (*n* = 1 per virus treatment; *n* = 2 from negative control tank). No specimens were available from the RHV-exposed group. Each tadpole was euthanized as previously described, immersed in 40 mL Poly/LEM fixative (Carson’s Modified Millonig’s Phosphate-buffered Formalin; Polysciences, Inc, Warrington, PA, USA) for 48 h, and then transferred to 70% ethanol. Subsequent dehydration, clearing, and paraffinization of the specimens followed standard histological procedures [75]. Serial sagittal sections, 4 or 5 µm thick were mounted on Fisherbrand ColorFrost Plus glass slides (Fisher Scientific, Pittsburgh, PA, USA). A portion of the 5 µm section slides underwent standard hematoxylin and eosin (H&E) staining as described in [76].

The presence of viral antigens in SVCV-exposed animals was investigated using an antibody-based immunohistochemistry (IHC) assay. Positive control tissue samples were obtained from whole koi confirmed to be infected with the NC2002 SVCV strain in a previous study [77]. Following dewaxing and rehydration of 5 µm transverse sections, antigen retrieval was performed by either of two methods: microwaving (0.1 M citrate buffer, pH 6.0 for 6 min at medium power) or protease digestion (0.05% protease XIV in Tris-buffered saline [TBS, 0.05 M Tris-HCl, 0.15 M NaCl, pH 7.6] for 15 min at 37 °C). Endogenous alkaline phosphatase was quenched with BLOXALL blocking solution (Vector Laboratories, Newark, CA, USA) for 10 min. Two custom anti-SVCV polyclonal antibodies [78] generated from rabbits receiving 2 subcutaneous injections (primary and 4-week booster) of a temperature-inactivated purified SVCV (Canadian strain HHOcarp06-genotype Ia) preparation were graciously provided by the Pathobiology Department at the University of Guelph (Guelph, ON, Canada). The concentrated antibody solutions were diluted 1:400 and 1:800 in TBS to determine the optimal antibody concentration and hybridized to sections for 30 min. A MACH 4 Universal AP-Polymer Kit and Vulcan Fast Red substrate-chromogen (Biocare Medical, Pleasant Hill, CA, USA) were used to detect antibody binding sites according to the manufacturer’s instructions. Sections were counterstained with CAT hematoxylin (Biocare Medical). Negative control samples consisted of sections from non-SVCV-infected koi or sections in which the primary antibody was replaced with TBS.

Histopathology in SVCV-exposed animals was also investigated using a molecular probe-based chromogenic in situ hybridization (ISH) approach to detect SVCV RNA. A custom RNAscope probe was designed to bind to RNA encoding the SVCV N-protein (Advanced Cell Diagnostics [ACD] Catalog# 1207811-C1, Newark, CA, USA). Samples were processed at the WFRC and PSF histopathology laboratories with the 2.5 HD Reagent–Red staining kit as described previously [79]. The Human Tbp gene probe (ACD Catalog# 314291) served as the ISH assay negative control probe. Sections (4 µm in thickness) of SVCV-infected koi from a previous study [77] and naïve koi were screened to establish the assay’s analytical performance. Sections were baked for 1 h at 60 °C, dewaxed and rehydrated through a graded ethanol series to deionized water, and then treated with hydrogen peroxide for 10 min to inactivate endogenous peroxidase. After boiling for 15 min in target retrieval reagents, sections were digested with proteases for 30 min at 40 °C. SVCV and Tbp probes were hybridized for 2 h at 40 °C and then sections were incubated in a series of amplifying reagents (AMP 1 through 6) according to the manufacturer’s recommended incubation times and temperatures. Signals were detected with Fast Red for 10 min, counterstained with 50% Gill’s hematoxylin, and mounted with Faramount (Agilent Dako, Santa Clara, CA, USA). A positive SVCV signal, using red chromogenic detection, was described as visualization of a red stain or dye in a tissue/cell. All stained tadpole slide sets were examined as blind sample sets under brightfield microscopy for confirmatory detection of SVCV and/or any tissue anomalies.

### 2.6. Fish and Tadpole Cohabitation Challenge 

**Figure 1:**
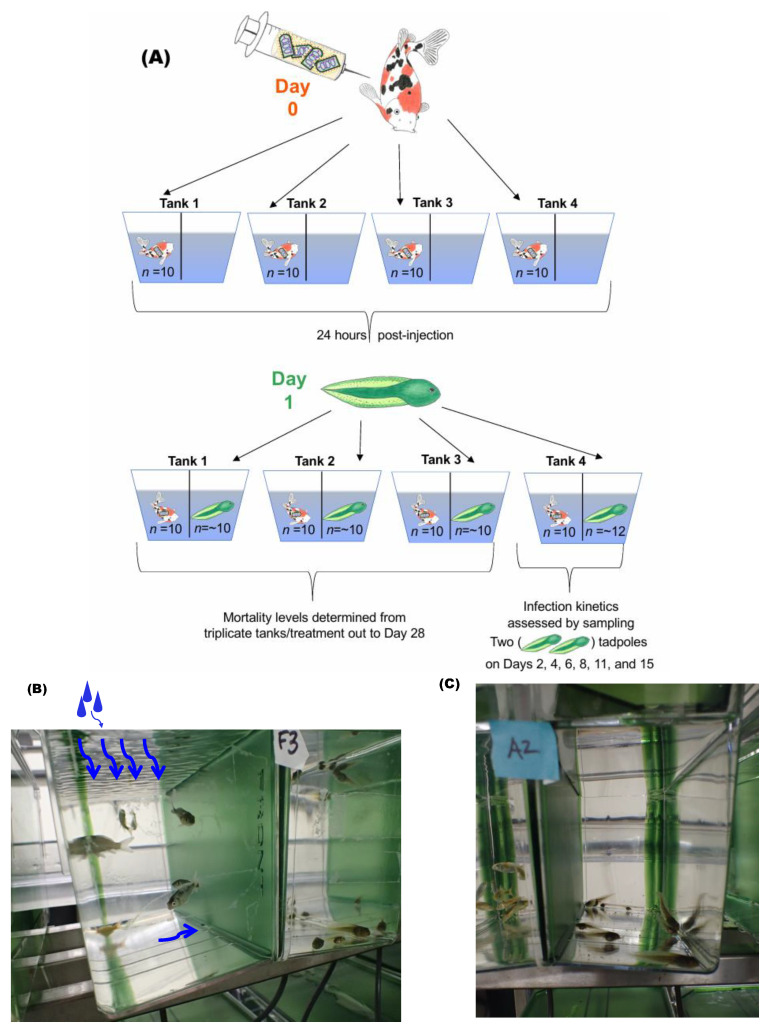
**Koi fish and Pacific tree frog cohabitation virus challenge.** (**A**) Schematic illustration of the cohabitation experiment for a single spring viremia of carp virus (SVCV) treatment group from Day 0 to the end of the experiment (Day 28). Each koi *Cyprinus rubrofuscus* received an intraperitoneal injection of an SVCV strain at a dose ranging from 4.5 × 10^6^ to 1.4 × 10^7^ plaque-forming units (PFU)/fish on Day 0 and naïve Pacific tree frog *Pseudacris regilla* tadpoles were placed on the other side of the tank on Day 1. Animals were distributed into 4 replicate tanks for each virus strain tested with 3 tanks designated for monitoring mortality (*n =* 9–11 koi and 8–11 tadpoles/tank) and 1 tank with 12 tadpoles for evaluating infection kinetics. Targeted sampling of 2 tadpoles per timepoint tank was conducted 2, 4, 6, 8, 11, and 15 days after the experiment started. (**B**) Day 7 image of one 8 L tank displaying SVCV-injected koi on the left side and naïve tadpoles on the other side of the tank. Water flow dynamics of the cohabitation challenge are highlighted. Incoming freshwater was dribbled on the surface of the koi side at the front of the tank (multiple blue arrows). There was a small gap at the bottom of the barrier (single blue arrow) for water, potentially containing shed virus from the koi, to flow over to the tadpole cohabitants. (**C**) Day 11 image of one tank displaying naïve tadpoles on the right side of the 8 L tank and mock-injected koi on the other side of the tank near the corner baffle.

A domestic stock of koi acquired from a distributor in Washington State was transferred to the WFRC BSL-2 wet lab and reared in 4-foot-wide circular tanks under the same water conditions and IACUC-approved animal care procedures described previously, except they were held at water temperatures of 18–19 °C. Every second day, the koi were fed a mixed diet of Hikari Gold mini-pellets (Kyorin Food Industries) and Classic Fry 1.5 mm floating pellets (Skretting, Tooele, UT, USA). On day 0 of the experiment, the koi were 2 months of age, with average weights of 0.86 g (0.41–1.33 g) and average lengths of 4.2 cm (3.5–4.6 cm). They were transferred to the aquatic BSL-3 laboratory to serve as the virus donors (progenitors) in the cohabitation challenge (Figure 1A). The fish were anesthetized in a 2 L water bath containing 0.12 g of MS-222 and 0.6 g of NaHCO_3_ and each koi received a 100 µL intraperitoneal injection of either SVCV 20040741 (4.5 × 10^6^ PFU/fish), SVCV NC2002 (4.9 × 10^6^ PFU/fish), SVCV RHV (7.8 × 10^6^ PFU/fish), SVCV P4-7 (5.3 × 10^6^ PFU/fish), SVCV Fijan (1.4 × 10^7^ PFU/fish), or M-10 (cell culture media). There were 9–11 donor koi/tank with 4 replicate tanks/treatment. After injection, each set of koi for every treatment was placed on one side of an 8 L tank divided in half by a barrier and monitored until fully recovered from the injection procedure (Figure 1B). 

Twenty-four hours after the koi were injected (Day 1), Pacific tree frog tadpoles from the same stock utilized in the immersion challenge were transferred from the BSL-2 wet lab stock tanks to the aquatic BSL-3 laboratory. The tadpoles were placed in the other half of the divided 8 L tanks *(n* = 8–11 tadpoles/tank) and served as the naïve recipients of virus shed from the virus-injected donor koi segregated on the other half of the tank (Figure 1C). Water flow tank dynamics for the cohabitation experiment consisted of incoming water being trickled at a rate of 0.087 L/min onto the water surface at the front of the tank on the koi side (Figure 1B). Water could flow from the koi side to the tadpole side through a gap at the bottom of the barrier and wastewater exited through two baffles located in the back outer corners on each side of the tank. The cohabitation challenge lasted 28 days with water temperatures held constant at 14.3–14.5 °C. Since the immersion and cohabitation experiments were run concurrently in the aquatic BSL-3 laboratory, the cohabitation water temperature rose to 16 °C on Day 22 during an unplanned 1.5 h shutdown of the inflow water system. The animals were monitored daily, fed every other day, and any observed dead or dying animals were removed from the tank daily. Each individual specimen was collected and stored as previously described in Section 2.4. Cumulative mortality in three replicate tanks per treatment was monitored to determine tadpole susceptibility to SVCV. Targeted sampling was conducted using animals in the fourth treatment tank (starting *n* = 12 tadpoles/tank) to qualitatively assess infection success of a naïve host (tadpoles) from a virus-donor host (koi). In this case, two tadpoles per tank were removed on days 2, 4, 6, 8, 11, and 15 post-koi injection. On designated sampling days, tadpoles that were displaying signs of disease (hemorrhages) were preferentially selected and euthanized. In the absence of clinical signs, animals were randomly selected for euthanasia and frozen until samples were processed.

### 2.7. Specimen Processing and Virus Detection

Subsets of the collected frozen specimens (salamanders, frog tadpoles, or koi) from each challenge experiment were thawed and homogenized in chilled (4 °C) MEM containing antibiotics at ratios of 1:5 or 1:8 based on body weight using the same procedures detailed in a previous study [77]. Specimens were selected such that each subset included animals from each of the replicate tanks/treatment. After homogenization, each clarified supernatant of a specimen was screened for SVCV by two methodologies: plaque assay to determine the concentration of recovered viable virus and reverse transcription quantitative polymerase chain reaction (RT-qPCR) for molecular enumeration of SVCV N-gene copies. For the plaque assay, a 100 µL aliquot of clarified supernatant was further diluted, as in [70], except incubation temperatures were performed at 20 °C. The assay has a detection limit of approximately 100 PFU/g of tissue [80] and results were reported as log 10 PFU/g of tissue. For the RT-qPCR assay, total RNA was extracted from a separate 210 µL aliquot of the clarified supernatant using a Direct-zol RNA Miniprep kit according to the manufacturer’s (Zymo Research, Irvine, CA, USA) instructions. The extracted RNA was resuspended in 50 µL RNase-free water, quantified using a NanoDrop spectrophotometer (Thermo Scientific, USA), and frozen at −80 °C until RT-qPCR testing. Molecular testing of the amphibian tissue samples using the RT-qPCR Q2N assay was conducted as described in [81]. Virus titer was determined using the standard curve method and expressed as equivalent plasmid copies (EPC) per g tissue. 

### 2.8. Data and Statistical Analyses

Cumulative percent mortality (cpm) was calculated for each virus challenge experiment and used as a proxy for amphibian species susceptibility to SVCV (cpm = mean (# dead or moribund amphibian/total number of animals exposed × 100 per replicate)). Normality of the residuals was evaluated using Shapiro–Wilk and Kolmogorov–Smirnov tests. The number of salamanders available for the injection challenge study was insufficient to meet conditions for normality testing, though statistical tests are still robust when small departures from normality occur. In this case, a contingency table of mortality data for each treatment group was constructed and a Fisher’s exact test was performed to determine if significant differences in the two outcomes (death or survival) existed between salamanders from the mock or virus treatment groups. A paired *t*-test was used to determine whether statistically significant differences existed between the viable virus titer recovered from each salamander relative to the challenge dose it received. 

The cumulative mortality results for each treatment group within the immersion and cohabitation challenge studies were assessed for significant differences with the one-factor analysis of variance (1-way ANOVAs) test. If the raw data were normally distributed, an ordinary ANOVA test was conducted followed by the Tukey–Kramer post hoc test. Raw data sets lacking a normal distribution were evaluated with the Brown–Forsythe ANOVA and Dunnett T3 tests. Viable virus and viral RNA concentrations for tadpoles collected at discrete intervals (timepoints) after immersion exposure were compared by a 2-way ANOVA mixed model test with Bonferroni multiple-comparison post-tests performed if applicable. Virus titers of tadpoles sampled at intervals during cohabitation were averaged for each treatment. No statistical analyses were performed on these values because only two tadpoles were sampled per timepoint for each treatment. 

Kaplan–Meier survival analysis was performed for all challenge studies with the Mantel–Cox log rank statistic used to test for significant differences between the survival curve and survival time of two or more treatments. Pairwise comparisons of *p*-values were based on Bonferroni-corrected thresholds. Intra-study pairwise comparisons were performed between virus treatment groups and between the virus and mock treatment groups. Treatment groups with replicate data sets that lacked homogeneity of variance were not pooled and the raw data survival curves were presented instead. 

Tests for normality/homogeneity of variances, Fisher’s exact tests, paired *t*-tests, 1-way ANOVA, Tukey or Bennett post-tests, and survival analyses utilized the GraphPad Prism software (version 10.1.1) with significant relationships concluded for all comparisons at *p*-values ≤ 0.05.

## 3. Results

### 3.1. Susceptibility of Larval Salamanders to SVCV after Injection

**Figure 2:**
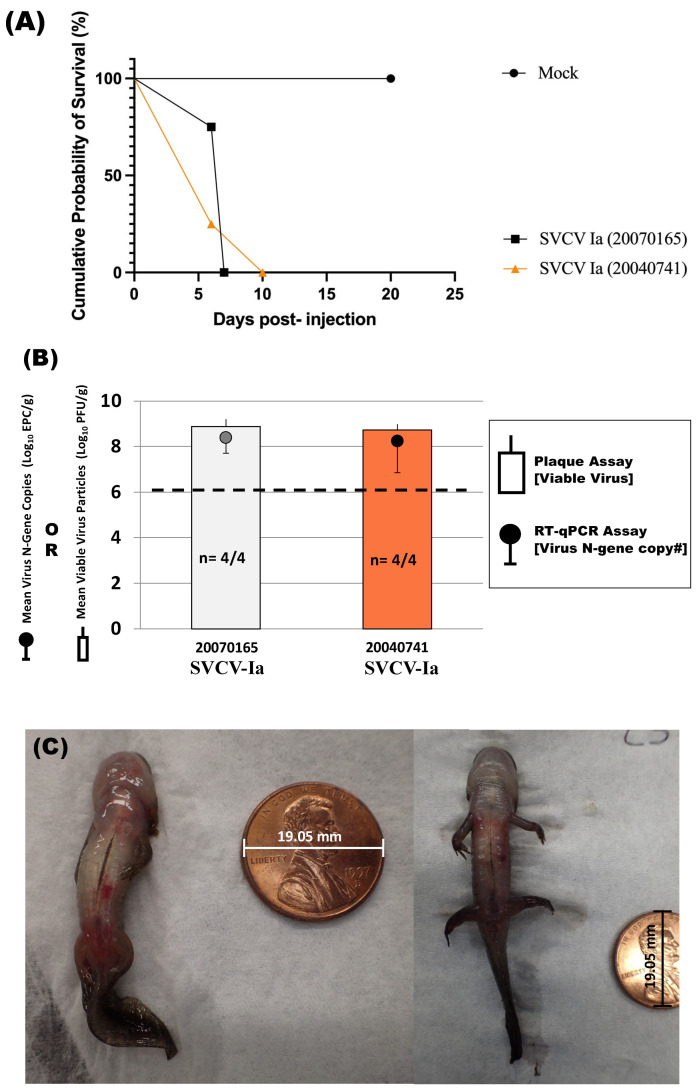
**Salamander response after exposure to SVCV by intraperitoneal injection.** (**A**) Cumulative probability survival curves for larval Western Long-toed salamanders *Ambystoma macrodactylum* exposed to SVCV Ia strains 20070165 or 20040741 or mock-inoculum. Animals were intraperitoneally injected on Day 0 and study was terminated on Day 20. Each curve represents the mean survival from 4 replicate tanks. (**B**) Viral load in salamanders that died after injection with SVCV. Samples of individual salamanders (average weight 1.1 g) were tested for viable virus by plaque assay (reported as log_10_ plaque-forming units (PFU)/g of tissue) and virus gene copies via RT-qPCR (reported as log_10_ equivalent plasmid copies (EPC)/g of tissue). Mock-exposed salamanders had no detectable levels of virus (see raw data citation below). The dashed line on the *y*-axis at log_10_ 10^6^ PFU/g denotes the viral dose each salamander received at the start of challenge. Error bars represent one standard deviation from the mean virus titer for each diagnostic test method. (**C**) Larval salamanders that died on Day 5 (SVCV 20070165, left panel) and Day 6 (SVCV 20040174, right panel) post-injection. Animals displayed external clinical signs of disease including extensive diffuse hemorrhage on the ventral skin, ascites, and petechial hemorrhages around the mouth and on the legs.

The cumulative mortality observed in salamanders exposed to the two SVCV Ia strains was 100% with all eight animals dead within 10 days of virus exposure (Table 2, Figure 2). Significant differences in cumulative mortality (two-sided *p* = 0.0286) and survival probability (*p* ≤ 0.0100, Figure 2A) were found between the virus-exposed and the mock-exposed salamanders. Salamanders injected with isolate 20070165 all died by day 7 post-exposure (median survival time = 7 days). The last remaining salamander in the group exposed to strain 20040174 died on day 10 post-injection (median survival time = 6 days). No significant difference was observed between the survival probabilities (*p* = 0.7441) of salamanders exposed to the two Ia strains of SVCV. The mock-exposed salamanders were alive and apparently healthy out to 19 days post-exposure (dpe). On Day 20, one of these salamanders escaped from the tank to the lab outflow drain. As a result, the trial was terminated and the remaining salamanders were euthanized.

The mock-exposed salamanders tested negative for virus by plaque assay and RT-qPCR analyses (*n* = 3, see raw data citation below). Virus was detected by both methods in 100% of the virus-exposed salamanders that died (*n* = 4/4 per Ia strain) (Figure 2B). Mean viable virus titers recovered from virus-exposed salamanders were 10^8.8^ PFU/g (20070165: 10^8.7^ to 10^9.4^ PFU/g, SD = 10^0.32^) and 10^8.7^ PFU/g (20040174: 10^8.4^ to 10^9.0^ PFU/g, SD = 10^0.25^). The RT-qPCR assay detected comparable levels of virus in the same salamanders: 20070165, 10^8.4^ EPC/g (10^7.5^ to 10^9.1^ EPC/g, SD = 10^0.68^) and 20040741, 10^8.2^ EPC/g (10^6.2^ to 10^9.3^ EPC/g, SD = 10^1.39^). The geometric means of recovered viable virus concentrations of 10^8.8^ PFU/g (20070165) and 10^8.7^ PFU/g (20040174) were 2-fold higher (Figure 2B) and significantly different (*p* = 0.0008 and 0.0003) than the corresponding challenge dose. The majority of the virus-exposed salamanders that died (*n* = 7/8) manifested clinical signs of disease including extensive ventral skin hemorrhages, ascites, and hemorrhaging around the mouth and on the legs (Figure 2C).

### 3.2. Susceptibility of Pacific Tree Frog Tadpoles after Exposure to SVCV by Immersion

#### 3.2.1. Mortality/Survival of Tadpoles during the 28-Day Challenge

**Figure 3:**
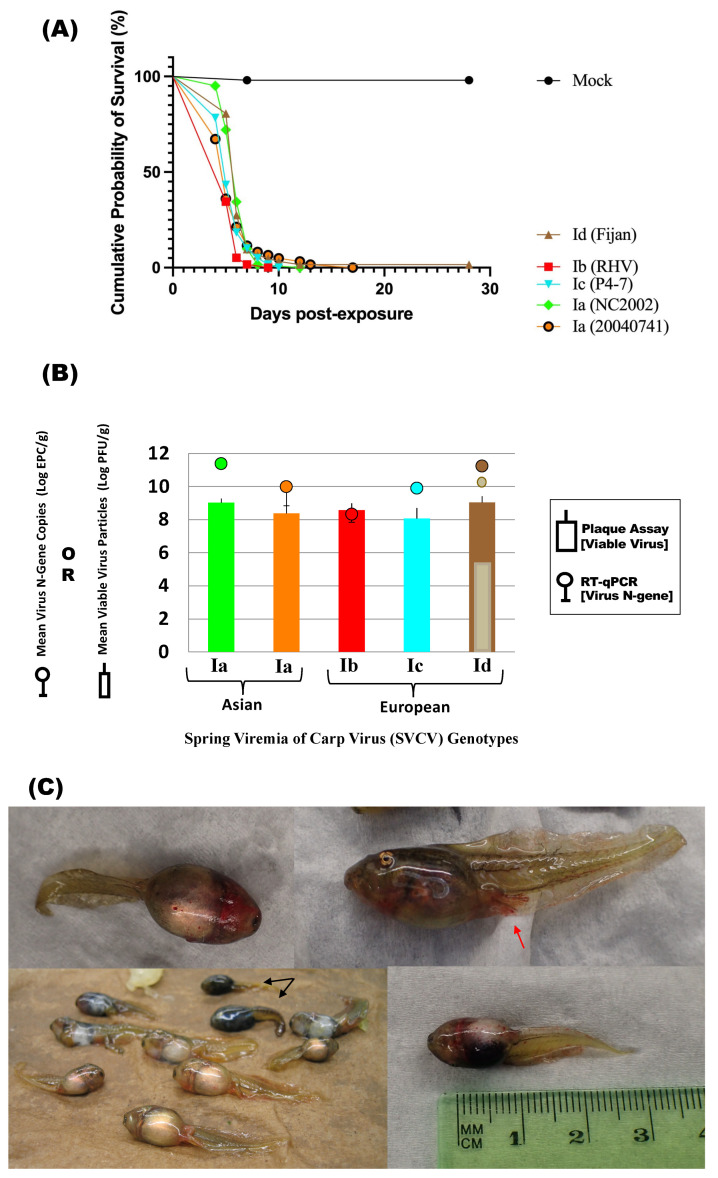

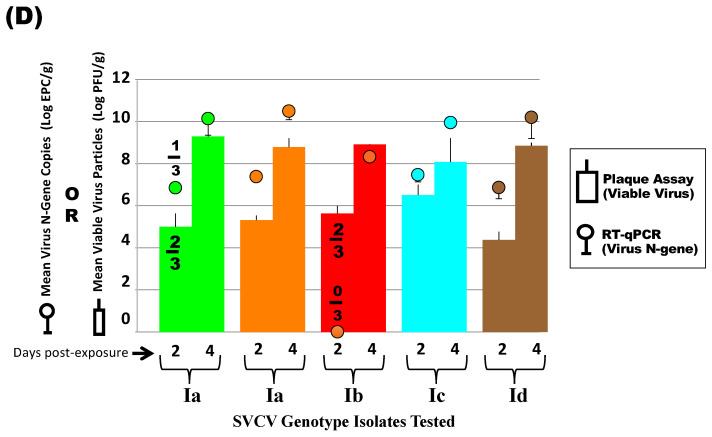
**Tadpole response after immersion exposure to SVCV.** (**A**) Cumulative survival of Pacific treefrog *Pseudacris regilla* tadpoles after immersion exposure (1 × 10^5^ PFU/L) to spring viremia of carp virus (SVCV) strains: NC2002 (Ia), 20040741 (Ia), RHV (Ib), P4-7 (Ic), or Fijan (Id). (**B**) SVCV titer in tissue homogenates collected from tadpoles that died following virus exposure. Titer was determined by plaque assay (PFU/g) or RT-qPCR (equivalent plasmid copies (EPC/g). Error bars represent one standard deviation from the geometric mean virus titer. Virus titer for the sole survivor from the Fijan treatment group is displayed as an additional inset bar and circle (colored in a light brown). All mock-exposed tadpoles screened for virus tested negative (see raw data citation below). (**C**) Clinical signs of disease evident in tadpoles that died include diffuse petechial hemorrhages on the fin tail, extensive ventral hemorrhages, limb bud inflammation (red arrow), and distended abdomens. Tadpoles with no external lesions are denoted with black arrows. (**D**) Virus titer in tadpoles sampled on days 2 and 4 following exposure. Virus prevalence ratios (number of virus-positive tadpoles/number of tadpoles tested) are presented for the plaque assay (in bars) and RT-qPCR (above circles). The molecular titers for isolate RHV are illustrated as light red circles to distinguish them from the RHV viable virus concentrations (red bars). Prevalence ratios from each test method are only shown if they differ from one another. If no prevalence ratio is shown, then all tadpoles tested positive (*n* = 3/3) for virus by both detection methods. Error bars represent one standard deviation from the geometric mean virus titer. An equivalent number of mock-exposed tadpoles (*n* = 3/timepoint, total *n* = 6) screened for virus on the same days by both methodologies tested negative (see raw data citation below).

Cumulative mortality of virus-exposed tadpoles (98–100%) was higher as compared to the mock-exposed tadpoles (2%), and significantly different (adjusted *p* < 0.0001) (Table 2, Figure 3A). No significant differences in mortality were found between groups exposed to SVCV (adjusted *p* ≥ 0.9598). Cumulative mortality ranged between 58 and 62 tadpoles/virus treatment group (total number of dead virus-exposed tadpoles = 301/302). 

Mortality onset was rapid in the virus-exposed tadpoles (Figure 3A). The median survival time ranged from 5 days (20040741 (Ia), RHV (Ib), P4-7 (Ic)) to 6 days (NC2002 (Ia), Fijan (Id)). By day 17 post-exposure, only a single tadpole from the Fijan virus (Id) strain treatment group was alive and remained so until the end of challenge (1.61% cumulative probability of survival). The cumulative survival probability of 98% in the negative control group was due to the death of one tadpole on day 7 post-exposure, (Figure 3A). The survival curves of the mock and virus-exposed tadpole treatment groups were significantly different (*p* < 0.0001). 

A subset of the mock-exposed or dead tadpole specimens collected from each group were screened for virus using the PA and the RT-qPCR tests. The mock-exposed tadpoles tested negative with both assays (*n* = 4, one dead and three survivors (one from each replicate tank)) (see raw data citation below). Virus prevalence was 100% in the 16 individual or pooled samples collected from tadpoles that died following virus exposure. These samples consisted of three specimens per group from each of the SVCV treatment groups Ia (NC2002), Ib, Ic, and Id plus four from the Ia (20040741) group. Viable virus was detected with geometric mean titers of 10^8.1^ to 10^9.0^ PFU/g (SD = 0.2 to 0.6) and viral RNA copy numbers of 10^8.3^ to 10^11.4^ EPC/g (SD = 0.3 to 1.2). Virus was also detected in the sole survivor of virus exposure (Id, 28 dpe) with virus titers of 10^5.6^ PFU/g by PA and 10^10.4^ EPC/g by RT-qPCR (Figure 3B). Some of the tadpoles died with no external lesions; however, most of the tadpoles exhibited clinical signs of disease which typically included diffuse petechial hemorrhages on the fin tail, diffuse hemorrhages on the ventral side of the body, limb bud inflammation, and distended abdomens (Figure 3C).

#### 3.2.2. Virus Prevalence at Discrete Timepoints after Tadpole Exposure to SVCV via Immersion

The mock-exposed tadpoles all survived and were collected out to the last proposed Day 18 timepoint (*n* = 23, see raw data citation below). Another two tadpoles were sacrificed on Day 8 and were used as the representative negative control specimens for histological examination. For the virus-exposed groups, specimens were collected as planned on days 2 and 4 post-exposure. Additional sampling planned for days 6, 8, 11, 15, and 18 post-virus exposure was not possible as no animals survived. For the mock-exposed tadpoles collected on days 2 and 4 post-exposure (*n* = 3/timepoint, total *n* = 6), no virus was detected by either PA or RT-qPCR testing (see raw data citation below). Virus was detected and quantified by at least one detection method (PA or RT-qPCR) for the virus-exposed tadpoles tested at the Day 2 and Day 4 timepoints (*n* = 3 tadpoles/strain/timepoint) (Figure 3D). On Day 2, virus prevalence was 33–67% in tadpoles exposed to isolate NC2002 (Ia) *(n* = 2/3 positive (PA); *n* = 1/3 positive (RT-qPCR)) versus 100% prevalence in tadpoles exposed to strain 20040174 (Ia), P4-7 (Ic), or Fijan (Id) (*n* = 3/3 positive per group). At the same timepoint, viable virus, but not viral RNA, was detected in 2/3 tadpoles tested after exposure to the RHV (Ib) isolate. Virus prevalence on Day 4 was 100% for all virus treatment group samples (3/3 per virus treatment) by both detection methods (Figure 3D). Petechial hemorrhages in the fins were observed for all tadpoles (*n* = 3 tadpoles/treatment) collected from the 20040741 (Ia) and RHV (Ib) treatment groups on Day 4. One tadpole from the P4-7 (Ic) treatment displayed minor clinical signs of disease. 

The average titer of viable virus detected by PA was lower on Day 2 (10^4.4^ to 10^6.5^ PFU/g, SD = 0.2 to 0.6) than on Day 4 (10^8.0^ to 10^9.3^ PFU/g, SD = 0.02 to 0.4), suggesting that SVCV was capable of attaching, entering, and then later replicating in tadpole host cells. Mean molecular titers of the SVCV genome in the tadpoles were higher than infectious virus concentrations for virus strains Ia, Ic, and Id on both Day 2 (10^6.8^ to 10^7.5^ EPC/g, SD = 0.01 to 0.5) and Day 4 (10^9.9^ to 10^10.5^ EPC/g, SD = 0.4 to 1.0). For the RHV (Ib) strain, no viral RNA was detected on Day 2 by RT-qPCR. On Day 4, the RHV isolate gene copy numbers (10^8.3^ EPC/g (SD = 0.1) were slightly lower than the viable virus concentration (10^8.9^ PFU/g, SD = 0.02) (Figure 3D). Inter-strain comparisons of virus titers for the two timepoints showed that for all SVCV isolates, tadpoles on Day 4 had viable virus concentrations that were significantly higher (adjusted *p* values ≤ 0.0054) than on Day 2, indicating that within host-replication occurred. With the exception of the RHV group of tadpoles, the same result was observed with viral RNA titers (adjusted *p* values < 0.0001). Since viral RNA was only detected on Day 4 for the RHV strain, no statistical comparisons could be performed. 

#### 3.2.3. Histopathological Examination of Tadpoles Exposed to Virus via Immersion

Positive control tissue sections from SVCV NC2002-infected koi tested negative with the immunohistochemistry (IHC) assay and positive with the in situ hybridization (ISH) assay. ISH testing of negative control tissue from mock-exposed koi produced the expected negative results. The IHC assay was not pursued further and tadpole tissue sections (*n* = 6, two mock-exposed and four SVCV-exposed tadpoles) were evaluated for SVCV RNA detection with the ISH method. For the H&E stained slide set, two of the four SVCV (strains 20040741 and P4-7) exposed tadpoles had evidence of hepatocellular degeneration with or without necrosis as compared to the other two SVCV (strains NC2002 and Fijan) exposed tadpoles and one mock-treated tadpole that had indications of hepatocellular vacuolation. No significant tissue anomalies were noted during evaluation of the other mock-exposed tadpole. Because only one H&E stained slide was available per animal to evaluate tissue morphology and lesions potentially attributable to viral exposure, or lack thereof, assessment of histopathological outcomes from the H&E stained slide set were limited. Thus, the H&E stained slides were used primarily to identify the specific tissues evaluated within each slide per animal for comparison with the ISH assay stained slides. 

**Figure 4:**
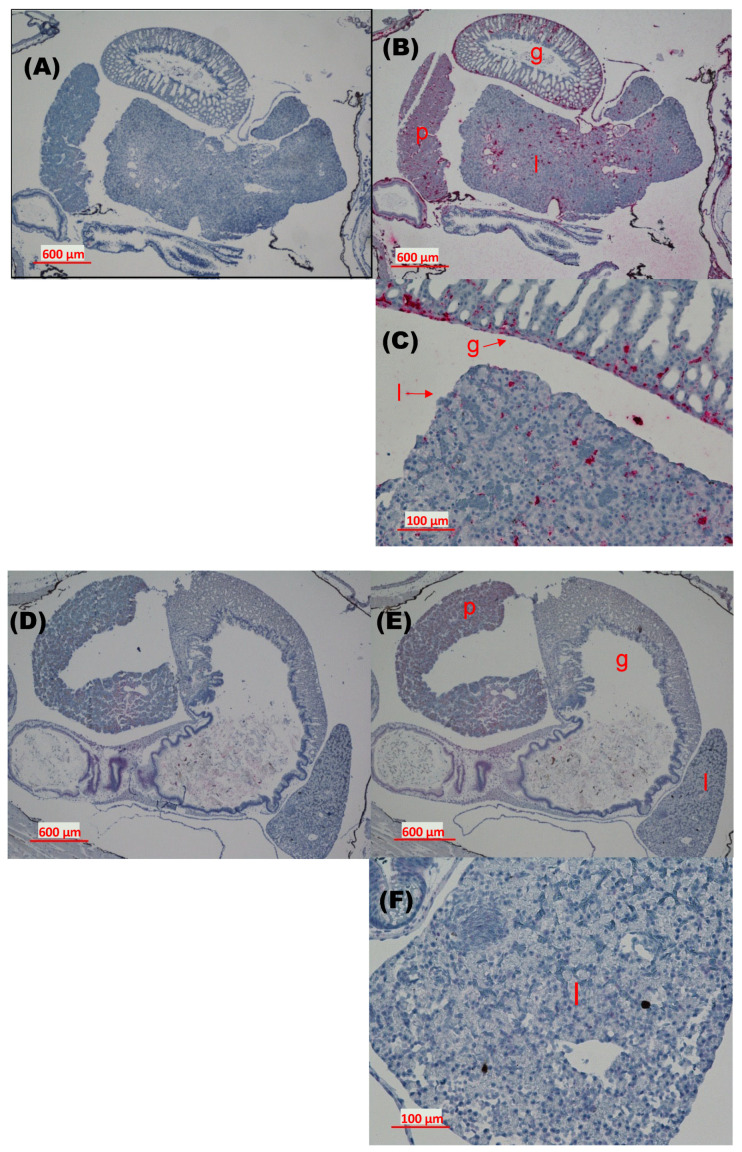
**In situ hybridization (ISH) of tissue sections from tadpoles exposed to SVCV or mock treatments by immersion**. Tissue sections (**A**–**C**) of a Pacific tree frog *Pseudacris regilla* tadpole collected 7 days after exposure to the SVCV strain 20040741 (Ia) and hybridized with the ISH negative control probe (**A**), or the SVCV-specific probe (**B**,**C**). Multiple foci of red SVCV-positive cells were evident within the gastrointestinal tract digesta (g), liver (l), and pancreas (p) ((**B**), 4× magnification) as well as in the submucosa of the gastrointestinal tract (g arrow) and in the liver parenchyma (l arrow) ((**C**), 20× magnification of panel (**B**)). Tissue sections (**D**–**F**) of a mock-exposed tadpole sampled on Day 8 post-exposure were stained with the ISH background stain ((**D**), 4× magnification) or with the SVCV-specific probe (**E**,**F**), with no SVCV-specific red staining visualized in the organs ((**E**), 4× magnification). Blue–black-colored cell aggregates were present in the liver ((**F**), 20× magnification of panel (**E**)).

Widely dispersed bright red staining indicative of SVCV probe hybridization was observed in organs of virus-exposed tadpoles tested with the ISH assay (Figure 4). The intensity of the red stain was consistent with the presence of relatively high virus titers in tissues. The staining pattern displayed in tissue sections from the tadpole exposed to SVCV strain 20040741 (Ia) was representative of that found in tadpoles exposed to isolates NC2002, P4-7, or Fijan (Figure 4B,C). In all cases, foci of red dye were dispersed on serosal surfaces (luminal and submucosal) of the gastrointestinal tract and within the parenchyma of the gastrointestinal tract, liver, and pancreas. Aggregates of SVCV-positive cells were evident in the submucosa of the gastrointestinal tract and liver parenchyma (Figure 4C). No red foci were observed when the same tissue section was screened with the negative control probe (Figure 4A). An artifactual light pink or purple diffuse coloration within the non-specific background stain was sometimes present in mock-exposed tadpole tissue sections of the liver, pancreas, and gastrointestinal tract (Figure 4D). However, the absence of red foci and the presence of blue–black aggregates following hybridization with the SVCV probe confirmed that the mock-exposed tadpoles tested negative for the virus (Figure 4E,F).

### 3.3. Susceptibility of Naïve Tadpoles after Cohabitation with SVCV-Infected Koi

#### 3.3.1. Mortality Levels of Koi Serving as the Virus Donors/Shedders

**Figure 5:**
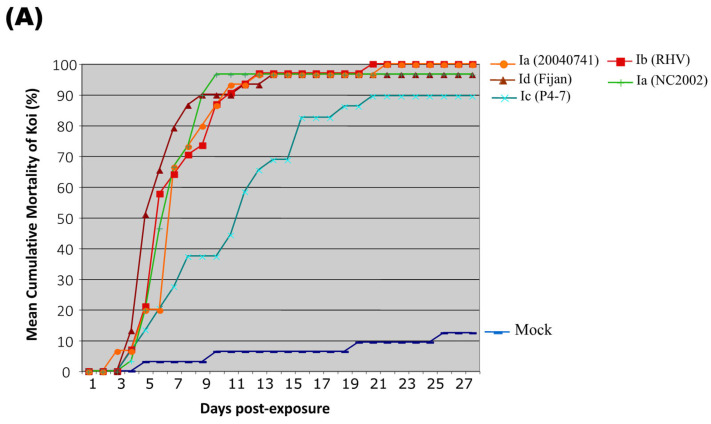

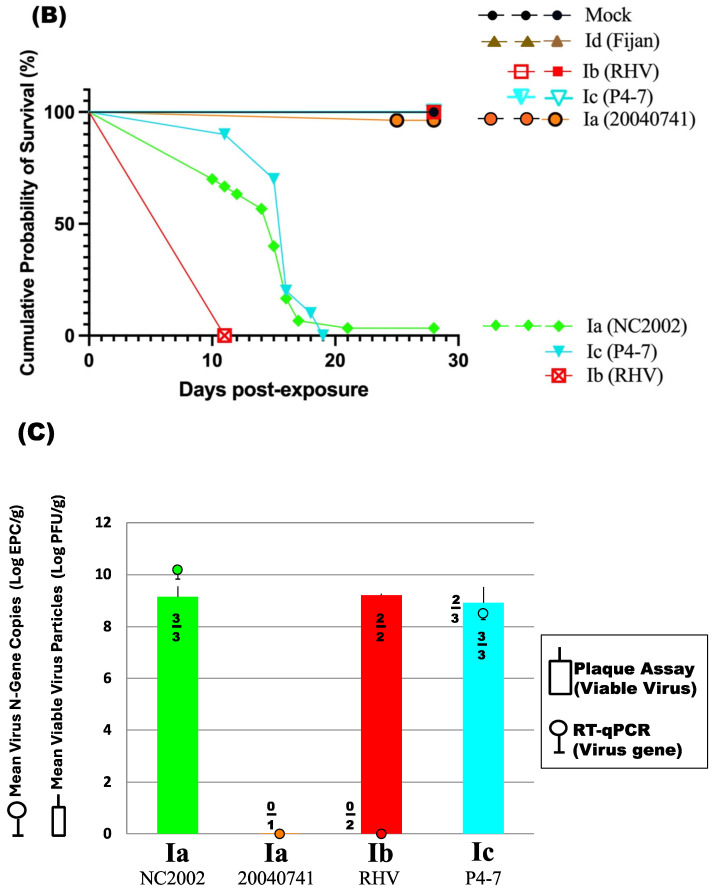

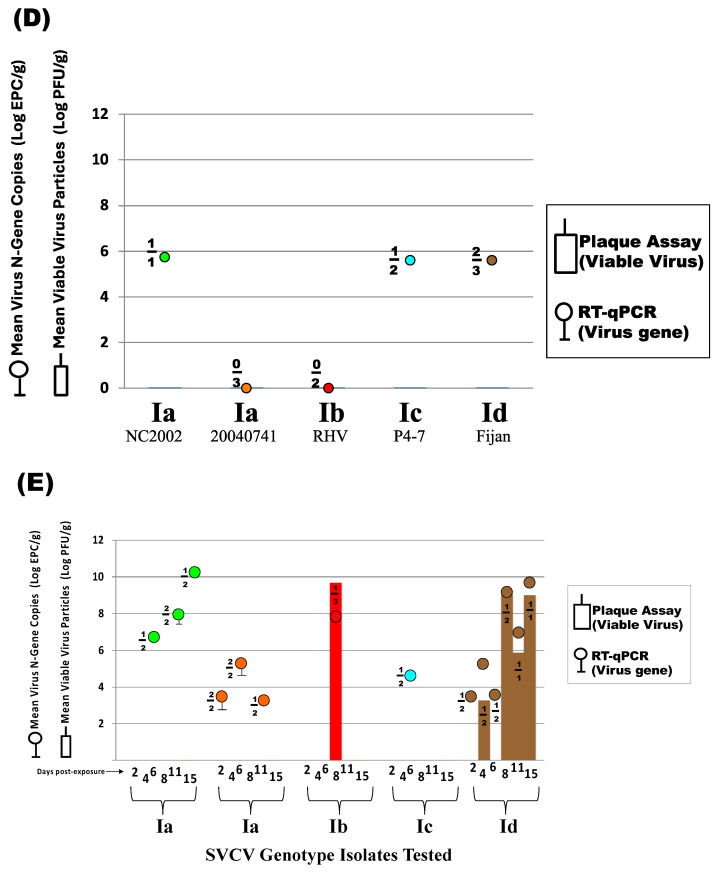

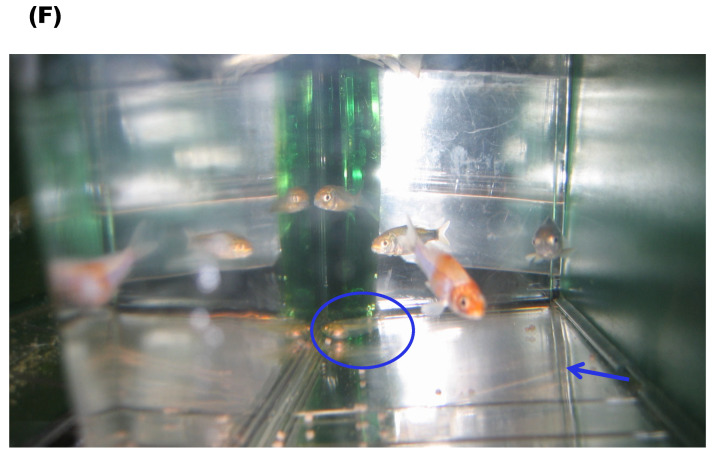
**Tadpole response after cohabitation with SVCV-infected koi.** (**A**) Cumulative mortality of koi *Cyprinus rubrofuscus* intraperitoneally injected with SVCV strains: NC2002 (Ia), 20040741 (Ia), RHV (Ib), P4-7 (Ic), or Fijan (Id), or sterile cell culture media (mock). (**B**) Survival probability curves for naïve Pacific treefrog *Pseudacris regilla* tadpoles spatially separated but cohabiting in the same tank as the SVCV-infected koi. Cumulative percent survival curves are shown for tadpoles from 3 replicate tanks for the mock, NC2002 (Ia), 20040741 (Ia), and Fijan (Id) treatment groups because there was little to no variation in survival between the triplicate tanks (displayed as three identically colored symbols). Percent survival curves of individual tanks were presented for the RHV (Ib) and P4-7 (Ic) treatment groups that displayed inconsistent levels of tadpole mortality among the replicate tanks. For both treatment groups, duplicate tanks with same survival levels are displayed as two symbols *(n* = 2) and a single replicate tank (*n* = 1) is shown as one symbol. (**C**) Viral load in tadpoles that died after cohabiting with SVCV-infected koi. No tadpoles died during cohabitation with Fijan (Id) infected koi. Virus prevalence (*n* = number of virus-positive dead tadpoles/number of tested) is presented as a ratio for the plaque assay (PA; inside the vertical bars) and the RT-qPCR (adjacent to the circles) tests. Prevalence ratios are only displayed if test results from the 2 assays differ. Error bars represent one standard deviation from the geometric mean virus titer. (**D**) Viral load in tadpoles surviving 28 days of cohabitation with virus-infected koi. Virus prevalence (*n* = number of virus-positive surviving tadpoles/number of tested) is presented as a ratio (adjacent to the circles) for the RT-qPCR tests. No viable virus was detected by plaque assay in the virus-exposed tadpoles still alive at the end of the cohabitation trial. Error bars represent one standard deviation from the geometric mean virus titer. The screened survivors (*n* = 3, one from each replicate tank) from the mock-exposed group tested negative for virus (see raw data citation below). (**E**) Viral load in cohabiting tadpoles sampled at timepoints 2, 4, 6, 8, 11, or 15 days post-exposure to SVCV shed from infected koi. Virus prevalence *(n* = number of virus-positive tadpoles/number tested at discrete intervals) is presented as a ratio for the plaque assay (PA; inside the vertical bars) and the RT-qPCR (adjacent to the circles) tests. Mock-exposed tadpoles (total *n* = 11, sampled at the same intervals) were screened by both methodologies and tested negative (see raw data citation below). (**F**) Image of tadpole RT-9 on the side of cohabitation timepoint tank housing RHV-injected koi. Tadpole RT-9 (outlined by blue circle) is suspected to have been small enough to swim through the gap (highlighted by blue arrow) in the barrier used to physically separate the tadpoles from the koi. Tadpole RT-9 died on Day 8, which was the earliest day of death for tadpoles from the cohabitation challenge and was the only tadpole *(n* = 3, 1 dead and 2 alive) collected that day from the RHV timepoint tank testing positive for virus.

Cumulative mortality in koi injected with SVCV ranged from 90 to 100% (*n* = 143 died/148 koi in virus treatment tanks). For the mock-injected koi, cumulative mortality was 10% (*n* = 4 died/31 koi in control treatment tanks). Koi fry injected with the Ic (P4-7) isolate had a cumulative mortality of 90% (*n* = 26/29) with the last death occurring 21 days after injection of the virus (Figure 5A). Virus strains Ia (NC2002) and Id (Fijan) induced 97% mortality (*n* = 29/30 and 28/29) that ended on Days 10 and 14, respectively. Cumulative mortality was 100% in groups of koi that received injections of Ia (20040741) or Ib (RHV) strains (*n* = 30/30 for both virus treatments). By Day 13, 97% of the koi from each group had died. The remaining koi survived until 21 (Ib) or 22 (Ia–20040741) days post-injection. The probability of survival of virus-injected koi was significantly less than the mock-exposed koi (*p* < 0.00001).

#### 3.3.2. Survival of Naïve Tadpoles Cohabiting with SVCV-Infected Koi during the 28-Day Challenge

Mortality results for virus-exposed tadpoles in the cohabitation study were inconsistent regardless of SVCV challenge strain (Table 2, Figure 5). No significant differences were detected in the mean mortality between virus treatment groups (*p* = 0.1114) due to the highly variable mortality outcomes for replicate tanks. Cohort tadpoles residing with Ia (NC2002) infected koi experienced the highest level of mortality in all the replicate tanks (97%, 29 dead/30 exposed) with animals dying between Days 10 and 21 (Table 2, Figure 5B). In contrast, low cumulative mortality was observed in tadpoles exposed to the other Ia (20040174) strain (4%, 1 dead/27 exposed) or the Id (Fijan) treatment group (0%, 0 dead/26 exposed). This all or nothing pattern of tadpole mortality was especially evident in the Ib (RHV) and Ic (P4-7) treatment groups (Figure 5B). In each case, 100% of the tadpoles in one replicate tank died whereas no mortality was observed in the other two replicate tanks (Table 2, Figure 5B). Mortality in the RHV-exposed group occurred over the course of one day (Day 11) when carcasses blocking the tadpole baffle restricted the rate of water outflow. Mortality in the P4-7-exposed group reached 100% by Day 19. All tadpoles cohabiting with the mock-exposed koi survived the 28-day challenge (total *n* = 27, Figure 5B).

A subset of tadpoles that died after cohabiting with virus-injected koi (*n* = 1–3 dead tadpoles tested from each virus treatment group, total *n* = 9) were screened for virus. SVCV was detected in 56% (RT-qPCR, 5/9 positive) to 89% (PA, 8/9 positive) of the virus-exposed tadpoles that died during the cohabitation study (Figure 5C). High titers of viable virus were found by PA in dead tadpoles (10^8.3^ to 10^9.5^ PFU/g) following their exposure to NC2002, RHV, or P4-7-infected koi. Comparable levels of viral RNA in the same animals were detected by RT-qPCR (10^8.3^ to 10^10.6^ EPC/g) with the exception of those exposed to RHV. In that RHV (Ib) group, none of the dead tadpoles tested positive by RT-qPCR. The single tadpole that died following exposure to 20040741 (Ia) tested negative using both assays. None of the tadpoles exposed to the Fijan (Id) strain were tested as mortality was not observed in this treatment group.

SVCV was detected in 0–36% of the tested tadpoles that survived virus exposure for 28 days in the cohabitation challenge (Figure 5D). Of the 11 animals tested, 4 were positive by RT-qPCR and all were negative by the PA method. The 4 tadpoles testing positive had virus titers of 10^5.7^ EPC/g (NC2002; sole survivor in tank with 100% cohort mortality), 10^5.6^ EPC/g (P4-7; survivor in one of the replicate tanks with 100% cohort survival), and 10^5.6^ EPC/g (Fijan; mean titer from 2 survivors in 2 replicate tanks with 100% cohort survival). Naïve tadpoles cohabited with the mock-exposed koi all survived and tested negative for virus (*n* = 3, 1 per replicate tank, see raw data citation below). 

#### 3.3.3. Virus Prevalence at Discrete Timepoints after Tadpole Exposure to SVCV via Cohabitation

SVCV prevalence was 7–28% in tadpoles live-sampled at discrete timepoints during the cohabitation study (Figure 5E). Of the 57 animals tested, sixteen tested positive for SVCV by RT-qPCR and viable virus was detected in four tadpoles by the PA method. Virus was initially detected by RT-qPCR at 2 dpe in the 20040174 and Fijan treatment groups and was still detected at 15 dpe in the NC2002 and Fijan-exposed tadpoles. Virus prevalence and titer determined by molecular testing was lowest in tadpoles exposed to RHV (0% (0/12); 0 EPC/g) or P4-7 (8.3% (1/12); 10^4.6^ EPC/g) and higher in the other three treatment groups: NC2002 (33% (4/12); 10^6.7–^10^10.4^ EPC/g), 20040174 (42% (5/12); 10^3.2^–10^5.1^ EPC/g), and Fijan (60% (6/10); 10^3.4^–10^10^ EPC/g). The four PA-positive tadpoles were collected from the Fijan group on days 4, 8, 11, and 15 and had viable virus concentrations ranging from 10^3.3^ to 10^9.1^ PFU/g. Clinical signs of disease were evident on three tadpoles collected from this group on Days 11 and 15. The absence of a single tadpole from the Fijan group was noted on Day 8 and was presumed to have been consumed by its cohorts after death or an initial miscount of tadpoles occurred at the start of the experiment. 

Only two tadpoles from the timepoint sampling tanks died during the cohabitation study. One was from the Fijan group that died on Day 10 and was too decomposed to analyze. The other dead sampled tadpole was from the RHV group and it had the highest viable virus concentration (10^9.7^ PFU/g; 10^7.9^ EPC/g) of any of the tadpoles tested from the cohabitation timepoint tanks and the earliest day of day death (Figure 5E). This tadpole cohabited with the donor koi on their side of the tank after it swam through the gap located at the bottom of the barrier dividing the tank on Day 3. The tadpole escapee, designated as specimen RT-9, co-mingled with RHV-injected koi and did not return to the tadpole-side of the tank (Figure 5F). The koi did not chase nor prey upon tadpole RT-9. The animal died on Day 8 with visible clinical signs of disease (petechial hemorrhages on the tail). 

Cumulative mortality in the virus-donor koi fish in the timepoint tank reached 100% (47/47) with death occurring between days 4 and 15 post-injection (see raw data citation below). One mock-exposed donor koi died on day 7 post-injection with no clinical signs of disease and tested negative for virus (see raw data citation below). None of the tadpoles (*n* = 0/12) cohabiting with mock-injected koi died and the tadpoles tested negative for SVCV using both detection methods (*n* = 0/11, one tadpole sample bag could not be located). 

## 4. Discussion

Amphibian species are facing the highest risk of extinction among the animal classes with emerging diseases being a primary cause of their population decline [58,59,82]. Our results suggest that salamanders and frogs likely have the capacity to become infected with, replicate, and/or transmit SVCV to other susceptible hosts in freshwater aquatic ecosystems, and as such, SVCV may pose yet another pathogen threat to the survival of amphibian populations in North America. 

### 4.1. Parameters Modulating SVCV Transmission, Disease Development, and Virus Detection of Amphibians after Experimental Exposure

Our study indicated causality between infection with SVCV and amphibian morbidity and mortality. Clinical signs of disease were evident in tadpoles and salamanders exposed to SVCV by intraperitoneal injection, immersion, or cohabitation. In the injection and immersion exposure challenges, mortality onset was rapid and cumulative mortalities reached nearly 100% with high titers of virus detected in all of the dead or moribund animals tested. No difference in cumulative mortality was detected between the SVCV isolates tested in salamanders and immersion-exposed tadpoles. If mortality is used as a correlate of virus virulence, then the results suggest that the fitness of all five isolates is similar in amphibian hosts. In contrast, the virulence of these same SVCV strains varied from low to high in koi fry exposed under the same challenge conditions [18,81]. Cumulative mortality ranged from 0 to 94% and typically peaked later (17–19 dpe) in koi relative to the tadpole groups in our study which displayed close to 100% mortality by 10 dpe. The kinetics of SVC disease development in pre-metamorphic frogs suggest that these animals are highly vulnerable to the virus. 

A productive infection was established by SVCV in amphibians through active replication in their tissues. For example, the titer of viable virus detected in salamanders that died following injection exposure was significantly higher than the challenge dose. In tadpoles exposed to SVCV by immersion, the average titer of the virus was higher at the later timepoint post-challenge. Gills are a primary portal of entry and initial site of replication for SVCV in fish followed by rapid dissemination of the virus to internal organs including the heart, liver, kidney, spleen, and gastrointestinal tract [83]. The presence of gills during early stages of frog development may transiently increase their susceptibility to SVCV. As frogs mature, their susceptibility to infection may decrease after the gills degenerate and respiratory gas exchange occurs through paired lungs. Localization of SVCV RNA in liver and gastrointestinal tissues of tadpoles parallels the establishment of viremia in internal organs of infected fish [15]. 

Comparing the cumulative mortality and infection kinetics across the three experiments revealed a dose-dependent pattern to virus transmission in amphibian populations. Exposure of the amphibians to higher virus levels in the injection and immersion challenges resulted in higher virus prevalence, more consistent mortality, and rapid onset of disease. The all or nothing pattern of mortality exhibited in replicate tanks of the cohabitation study suggested that one or more of the tank parameters was modulating virus transmission dynamics. Potential factors included water flow dynamics, tadpole proximity to virus-shedding progenitor fish, duration and titer of virus shed from progenitor hosts, and density of infected tadpole cohorts in the tanks. For each factor, the infectious virus dose was impacted, suggesting that it likely played a pivotal role in the mortality outcomes during the cohabitation challenge. In essence, if virus levels remained below a certain minimum threshold, cumulative mortality, virus prevalence, and virus titer were inconsistent among replicate groups. However, once an infectious dose threshold was exceeded, an epizootic event was observed among tadpole cohorts in a cohabitation tank.

The cohabitation challenge experiment was designed so that water would flow via a gap in the tank barrier from the compartment holding SVCV-infected koi to the one containing naïve tadpoles. The design did not account for potentially lower levels of circulating virus arising from effluent flowing out via the baffles located in the two back corners of a tank. The ramification of this design feature was illustrated by an experimental observation made on Day 11 for the RHV (Ib) tank in which all 10 tadpoles died on a single day. The baffle on the tadpole side of the tank had become semi-blocked by some of the carcasses, which slowed the water outflow (effluent) for a period of <24 h. The quantity of waterborne virus may have increased on the tadpole side of the tank during that short timeframe. Virus titers may have exceeded a minimum infectious dose and initiated an epizootic cascade that resulted in the 100% mortality observed in that single RHV tank. Some of the cohabited tadpoles tested were positive for SVCV by PA which is consistent with the presence of replicating virus. Because virus was only detected in a fraction of the tested animals, the variability in infection prevalence and relative titers of virus shed from individuals likely impacted virus transmission dynamics and disease outcomes. It could be argued that tank water became fouled while the baffle was blocked, leading to the rapid demise of the tadpoles in this replicate RHV tank. However, tadpoles are frequently reared in static water conditions with intermittent water exchanges occurring days apart. In our experiment, fresh water continued to flow into the tank. Further, the observed water clarity was still excellent (no turbidity or foul smell) on Day 11 when all the tadpoles died. In those treatment tanks for which no mortality was observed, the water outflow may have modulated the amount of circulating virus to levels below a minimum infectious dose required to initiate a disease event. 

The time span that virus-shedding koi were present during the cohabitation experiment also impacted the quantity of infectious virus particles in the tank water. Infected koi were not present for the entire duration of the cohabitation experiment which meant that the virus exposure dose was not consistent over time. Our animal welfare experimental protocols require that moribund or dead koi be immediately removed from the tank. In natural settings, virus-infected dead fish could potentially shed virus for days as the carcass slowly decomposes. Although the virus decay rate has not been reported, SVCV released into fresh water is known to remain infective for 4 weeks [15]. Most of the SVCV-infected koi died by day 14 post-injection which meant that relative concentration of infectious virus circulating in the tank was lower during the second half of the 28-day experiment. Cannibalism has been documented in tadpoles of some frog species and consumption of virus-laden necrotizing tissue could be another route of exposure for aquatic rhabdoviruses as has been previously demonstrated in experimental infections of amphibians with ranaviruses [84,85]. Replacement of dead or moribund koi with new virus donors or cohabitation with euthanized SVCV-infected fish/tadpole carcasses could be utilized to further explore these transmission pathways in future studies.

The fate of tadpole RT-9, the sole escapee that comingled with RHV-infected koi, underscored the unintended impacts of the tank experiment configuration. In our cohabitation experiment, the donor koi were physically separated from naïve tadpoles to mitigate the risk of koi preying on the tadpoles. Tadpole RT-9 died on sampling Day 8, with visible clinical signs of disease and without any injuries indicative of trauma due to koi aggression. It also had the highest viable virus concentration out of all the screened specimen samples from the cohabitation challenge. The rapid decline of tadpole RT-9, five days after moving into the infected koi compartment, along with its high virus titers, revealed the importance of spatial connectivity on the outcome of SVCV transmission occurring between sympatric virus-infected progenitor and naïve host species/populations. A key driver in the emergence of a disease in a novel host is contact rate (time) between the reservoir host and novel recipient host [86,87]. Unlike the tadpoles on the other side of the barrier, RT-9 was in close proximity to infected koi and was likely exposed to SVCV through direct (i.e., potential physical contact) and indirect (i.e., higher concentrations of waterborne virus shed from koi) horizontal transmission pathways. The infectious dose was at or above the minimum threshold required to establish an infection in RT-9 and below the threshold in the tadpoles sequestered on the other side of the barrier. Thus, an integrated cohabitation challenge model could be implemented in future experiments to test infection dynamics between virus-positive koi and naïve tadpoles when both direct and indirect horizontal transmission pathways are available.

Virus prevalence levels generated with the RT-qPCR test results were lower relative to those obtained with the plaque assay (PA) for populations exposed to the RHV strain. The false negative test results were particularly evident at earlier timepoints following virus exposure when virus levels were below the RT-qPCR assay’s limit of detection for this strain. Molecular-based diagnostic assays are generally considered more sensitive than cultivation-based assays for detection of viruses [88,89], which was the case in our study for the other SVCV genotype strains (Ia, Ic, and Id) tested. It was discovered later after the immersion and cohabitation experiments were conducted that the lower analytical sensitivity of the molecular assay for RHV was due to a single base mismatch in the reverse primer binding site on the virus genome [81]. Therefore, the RT-qPCR screening of tadpoles exposed to the RHV strain were more likely to give false negative results. This experimental caveat highlights the importance of using more than one test to determine the virus status of an animal, especially for screening a novel host species infected with an emerging virus variant. 

### 4.2. Factors Driving a Fish Rhabdovirus Cross-Species Transmission into an Amphibian Host

Successful cross-species infections occur if the trifecta of parameters involving the pathogen strain (aquatic rhabdovirus genetic variants), amphibian host (immunocompromised, diverse host pathiobiome), and environment (climate perturbations, water quality, shrinking freshwater bodies) coalesce under the ideal conditions for a host jump to occur. The primary abiotic and biotic factors that could facilitate cross-species transmission of SVCV into amphibian species include: an SVCV variant pool optimized for host jumping, propagated foreign species and/or an invasive species introduction with hitchhiker SVCV strains, temperature perturbations associated with climatic shifts, and SVCV co-infection with another microbe that induces pathology (secondary infections).

Phylogenetic analyses of rhabdoviruses on an evolutionary scale have concluded that ancestral rhabdovirus host jumps between fish and/or amphibians have occurred in the past [1,90]. Examples of inter-fish rhabdovirus cross-species transmission events include (1) an ancestral strain of infectious hematopoietic necrosis virus in sockeye salmon *Oncorhynchus nerka* diverging into a species-specific, highly virulent phenotype that primarily infects rainbow trout *O. mykiss*; (2) viral hemorrhagic septicemia virus (VHSV) genotype strains evolved and adapted generalist traits and infects over 70 fish species in both marine and freshwater ecosystems; and (3) a seatrout rhabdovirus, Egli virus, appears to have jumped into European perch *Perca fluviatilis* [91,92]. The genetic divergence of aquatic RNA viruses, including fish rhabdoviruses, in conjunction with the ever increasing anthropogenic stressors on amphibian populations present in the modern world suggests that cross-species transmission and establishment of an extant fish rhabdovirus, like SVCV, in amphibian species is probable [93,94]. The SVCV Ia genotypes, of Asian origin, have the highest mutation rate across genotypes and as such a more diverse pool of variants available for host-jumping [48].

#### 4.2.1. Aquatic Species That Are Propagated and/or Non-Native/Invasive Species Can Have Hitchhiker Pathogens That Facilitate Novel Host Jumps

Aquatic animals that are propagated for the pet trade, as a food source, or for use as laboratory test subjects are in many cases non-native and could harbor pathogens foreign to regional waterbodies near their rearing location [95,96,97,98]. If these non-native species are introduced and become established in a new aquatic ecosystem, they then can serve as disseminating reservoirs for pathogen hitchhikers, like SVCV, potentially resulting in pathogen spillover events in native species [99,100,101,102]. 

Multiple animal species that are widely distributed, such as koi and carp but also shrimp, ticks, and salamanders, could contribute to the spread of SVCV among amphibians. SVCV has been documented in moribund penaeid (marine) shrimp reared in Hawaii [29]. A global metatranscriptomic sequencing study of brine shrimp viruses identified 55 novel RNA viruses, including another rhabdovirus [103]. These researchers further supported the hypothesis that shrimp may serve as virus vectors by suggesting that cross-species transmission occurs via brine shrimp cysts (thick-shelled dormant embryos). Shrimp sharing the same aquatic spaces with fish and amphibian species could provide repetitive opportunities for RNA viruses, like SVCV, to jump between vertebrate and invertebrate hosts. A study examining the virus composition of the globally invasive Asian longhorned tick *Haemaphysalis longicornis* found 508 RNA viruses in ticks from a single province in China and, interestingly, a significant proportion were identified as or closely related to aquatic-animal viruses, especially sea lice rhabdoviruses [104]. The scientists hypothesized that birds preying upon virus-laden fish and shrimp transported them to land and the invasive ticks had a subsequent infectious contact event with the aquatic species carcass or bite-infected waterfowl. Multiple sightings of Chinese fire-bellied newts at the same two locations in Florida State suggest that the introduced ornamental newts have established breeding populations [62]. Given that the first detection of SVCV from an amphibian was in this same newt species imported into Florida [63] suggests that feral dissemination of both the non-native host (newt) and a hitchhiker pathogen (SVCV) could occur in North America.

#### 4.2.2. Climate Change Aids Cross-Species Virus Transmission Events

Polyculture of multiple aquatic species, fish, shrimp, and amphibians, etc., occurs globally and while having many environmental advantages, it is also a fertile niche for novel cross-species transmissions, especially as ambient rearing temperatures are forcibly shifted to warm water culture environments due to climate change [105,106,107,108,109,110]. Aquatic invasive species typically have generalist qualities, such as tolerance to broad thermal perturbations, that are attributed to their successful establishment and expansion into new regions [111,112,113]. Aquatic host range shifts are occurring due to climate/water temperature alterations and the consequences (rising water temperatures, droughts, flooding, stratification) will likely continue to cause new host habitat overlaps in both freshwater and saltwater environments [114,115,116]. Thus climate change may accelerate cross-species pathogen infections in new host species due to unfamiliar mingling among aquatic species, especially for the highly mutable rhabdoviruses with their known proclivity for host jumps [1,94,117,118]. The frequency of rabies rhabdovirus cross-species transmissions between novel bat hosts at the genus level were more dependent on host range overlaps versus how closely related the animal species were to one another [119], suggesting that for some rhabdovirus species, host proximity is a key factor in overcoming barriers leading to viral host shifts. A possible climate-based scenario supporting an SVCV transmission event comes from an early study analyzing potential habitat range shifts of 57 freshwater fish species in the continental United States due to climate change [120]. Two warm water adapted fish species, belonging to the family *Centrarchidae*, largemouth bass *Micropterus salmoides*, and bluegill *Lepomis macrochirus*, had the largest predicted range expansion. SVCV was later detected in these same fish species and for the first time from wild Centrarchids in 2008 at a reservoir in the State of Ohio [46]. The overall trend of warming waters concomitant with extreme water temperature fluctuations could facilitate viral entry into a naïve host and subsequent successful virus replication while a host is experiencing a non-normative host immune response from a temperature stressor [121]. An SVCV Ia variant, isolated from farmed grass carp *Ctenopharyngodon idella* in China, exhibited high virulence in a variety of fish species, grass carp, common carp, koi, and goldfish *Carassius auratus*, at unusually warm water temperatures ranging from 16 to 26 °C when experimentally infected [122]. Temperature tolerant aquatic species carrying their unique microbiota may interact with naïve species under thermal stress making them more vulnerable to foreign hitchhiker viruses [5,123]. The intersection of climate change induced habitat shifts, introduced/invasive species, and foreign pathogens may facilitate novel cross-species pathogen transmissions into native species (i.e., spillover). Our findings indicated that SVCV is capable of spillover infections into amphibian species native to the Pacific Northwest region of North America.

#### 4.2.3. SVCV Co-Infection: Is Pathology Induced by Opportunistic Secondary Microbial Infections?

Though we were able to demonstrate infection and disease in the tested amphibian species, and histologically demonstrate the presence of SVCV RNA in the tadpole tissues, we need to further examine the entire mechanism of pathology. A future study would need to have a temporal histological sample collection of the amphibians’ post-SVCV exposure and the creation of consecutive serial H&E sections at each timepoint for every animal tissue sample to evaluate disease pathogenesis in specific organs (e.g., liver and gastrointestinal tract) as the infection progressed. SVCV disease development in a novel amphibian host species may not be a single pathogen model of disease. SVCV infection could also be suppressing the amphibian’s immune system such that other opportunistic microbiota proliferate and contribute to pathology that leads to host death. A dominant pathogen (e.g., SVCV) may drive the infection, but additional microbial interactions (e.g., bacteria) may be needed for host pathogenesis, especially for aquatic species whose environment is a continuum of microbial-filled fluid [101,124]. A huge number of ornamental aquatic species are traded on an international scale and disease-associated morbidities/moralities from polymicrobial infections are frequently reported for these animals, which are likely transported under stressful conditions [125]. Members of an aquatic host’s microbial community can become pathogenic or altered under certain environmental conditions (e.g., sharing the same water during transport and rearing), or if an invading pathogen causes an imbalance in the infected host health, particularly in aquacultural settings with intensive rearing practices aimed at producing high animal yields [126,127,128,129]. 

There are numerous examples of rhabdoviruses being detected in fish that were concurrently infected with other known pathogens and/or subsequently experienced alterations in microbial compositions. Largemouth bass infected with both largemouth bass virus and hybrid snakehead rhabdovirus had the highest mortality over bass infected with just the individual viruses alone [130]. The farmed mandarin fish *Siniperca chuatsi* was co-infected with three different rhabdoviruses; two of the rhabdoviruses remain uncharacterized, and one, described as the Siniperca chuatsi rhabdovirus, continues to cause widespread outbreaks in multiple fish species and has evolved other unique variants [131,132]. Common carp experimentally infected with SVCV, via intraperitoneal injection at a non-lethal dose, had both internal and external mucosal microbiota imbalances (dysbiosis) that favored pathogenic over commensal bacteria, which suggested that secondary deleterious bacterial infections occurred post-SVCV infection [133,134]. Similarly, the gut microbiota of zebrafish *Danio rerio* infected with SVCV showed a loss in commensal bacteria and an increase in opportunistic bacteria commensurate with increased viral loads in target organs (spleen, kidney, and intestines) [135]. Carp edema virus (CEV) and SVCV were both found in common carp cultured at Ukrainian aquaculture facilities [136]. Investigators suggested that mortality outcomes could have been attributed to multifactorial interactions of various pathogens, CEV, SVCV, saprolegnia (fungus), and bacteria, detected in co-infected carp stocks. Likewise, amphibian diseases with severe outcomes historically attributed to a sole pathogen may need to be reconsidered if necrosis is the result of polymicrobial infections with a dominant pathogen under specific environmental conditions. Infections with multiple pathogens (ranavirus with secondary bacteria, ranavirus and helminth parasite, ranavirus and chytrid fungus, and dermocystid-ranavirus-chytrid fungus) have been reported in amphibian species [137,138,139,140]. Thus, the mechanism of pathology within SVCV-infected amphibians needs further examination to determine if SVCV is the sole etiological agent responsible for pathological outcomes or if other symbiotic microbes are also involved. 

## 5. Conclusions

Amphibian infection by a fish rhabdovirus, spring viremia of carp virus, appears feasible based on our preliminary studies. Infection was established following three routes of exposure—injection, immersion, and cohabitation—with the latter two mimicking natural virus transmission pathways. Transmission of the virus from infected koi and development of disease in cohabiting naïve tadpoles expanded the known host range of SVCV and illustrated its potential for cross-species transmission. Infection, in most cases, led to clinical signs, disease, and mortality indicating that these larval amphibian species, Western Long toed salamanders and Pacific tree frogs, can be replicative hosts of SVCV. Confirmatory transmission occurred with virus strains from all four SVCV genotypes. Virus was recovered from exposed animals and detected by three methodologies (plaque assay, RT-qPCR, and/or ISH) which confirmed that viable virus and viral RNA was present in moribund, dead, and surviving animals. Recovered virus concentrations from individual animals, was, in most cases, higher later in the course of the infection, indicating that replication had occurred in these novel amphibian hosts, contributed to clinical disease signs, and was likely the primary driver causing the death of infected amphibians. The presence of virus out to 15 or 28 days post-SVCV exposure in the surviving amphibians suggests they may have subclinical infections and could serve as carriers of the virus. Collectively, these data suggest that SVCV is a potential biothreat to amphibian species and support the hypothesis that a cross-species host jump of SVCV from a fish to an amphibian host species could occur. To date, all other vertebrate classes, except amphibians, have animals shown to be vulnerable to rhabdoviruses [2,6]. Our findings here indicate that all major classes of vertebrates, including amphibians, have species that are susceptible to rhabdovirus infections.

## Figures and Tables

**Table 1:** Spring viremia of carp virus isolates utilized in susceptibility experiments of amphibians.

Genotype	Isolate/Strain Identifier	Host Origin (Country Detected in)	Collection Year	Isolate Host (Fish Species)	Reference [Citation]
Ia	20070165	Asia (China)	2007	Grass Carp (*Ctenopharyngodon idella*)	[18]
Ia	20040741	Asia (China)	2003	Common Carp (*Cyprinus carpio)*	[72]
Ia	NC2002	Asia (USA)	2002	Koi (*Cyprinus rubrofuscus*)	[36]
Ib	RHV	Europe (Ukraine)	1989	Rainbow trout (*Oncorhynchus mykiss*)	[43]
Ic	P4-7 (or P4)	Europe (Russia)	1983	Common Carp	[43]
Id	Fijan (or S30)	Europe (Yugoslavia)	1971	Common Carp	[33]

**Table 2:** Cumulative mortality of native amphibians after exposure to spring viremia of carp virus (SVCV) in three challenge experiments.

Virus Challenge Exposure Method	Amphibian Species (Life Stage)	Treatment Group SVCV Genotype (Origin)	SVCV Isolate	Cumulative Mortality ^a^	Standard Deviation	Mortality in Replicate Tanks (#Deaths/# Animals Tested)
Injection	Long-toed salamander (larval to metamorph)	Mock	Neg Control	0%	±0%	(0/1, 0/1, 0/1, 0/1)
		Ia (Asia)	20070165	100%	±0%	(1/1, 1/1, 1/1, 1/1)
		Ia (Asia)	20040741	100%	±0%	(1/1, 1/1, 1/1, 1/1)
						
Immersion	Pacific tree frog (tadpoles)	Mock	Neg Control	2%	±4%	(1/16, 0/17, 0/18)
		Ia (Asia)	20040741	100%	±0%	(20/20, 21/21, 20/20)
		Ia (Asia)	NC2002	100%	±0%	(20/20, 23/23, 18/18)
		lb (Europe)	RHV	100%	±0%	(20/20, 20/20, 18/18)
		Ic (Europe)	P4-7	100%	±0%	(18/18, 22/22, 20/20)
		ld (Europe)	Fijan	98%	±3%	(21/22, 19/19, 21/21)
						
Cohabitation (with koi virus-donors)	Pacific tree frog (tadpoles)	Mock	Neg Control	0%	±0%	(0/9, 0/10, 0/8)
		Ia (Asia)	20040741	3%	±6%	(0/10, 1/9, 0/8)
		Ia (Asia)	NC2002	97%	±6%	(9/10, 10/10, 10/10)
		lb (Europe)	RHV	33%	±58%	(0/11, 0/10, 10/10)
		Ic (Europe)	P4-7	33%	±58%	(0/10, 0/11, 10/10)
		ld (Europe)	Fijan	0%	±0%	(0/8, 0/10, 0/8)
						

^a^ Final cumulative percent mortality calculated as the mean total mortality experienced by the tested amphibians in individual replicate tanks for each treatment group.

## Data Availability

The USGS raw data release for this research study can be located at https://doi.org/10.5066/P17AZUD2. The citation for this published data set is Emmenegger, E.J., 2024, Spring Viremia of Carp Virus (SVCV) Infection Trials of Pacific Northwest Amphibians: U.S. Geological Survey data release, https://doi.org/10.5066/P17AZUD2.
